# Mining the mutanome: developing highly personalized Immunotherapies based on mutational analysis of tumors

**DOI:** 10.1186/2051-1426-1-11

**Published:** 2013-07-29

**Authors:** Willem W Overwijk, Ena Wang, Francesco M Marincola, Hans-Georg Rammensee, Nicholas P Restifo

**Affiliations:** 1 Department of Melanoma Medical Oncology, The University of Texas M.D, Anderson Cancer Center, Houston, TX, USA; 2 Infectious Disease and Immunogenetics Section, Department of Transfusion Medicine, Clinical Center, National Institutes of Health, Bethesda, MD, USA; 3 Sidra Medical and Research Centre, Doha, Qatar; 4 Department of Immunology, Institute for Cell Biology, University of Tübingen, Tübingen, Germany; 5 Center for Cancer Research, National Cancer Institute, National Institutes of Health, Bethesda, MD, USA; 6 NIH Center for Regenerative Medicine, National Institutes of Health, Bethesda, MD, USA

**Keywords:** Mutanome, Mutation, Neoantigen, Peptide epitope, T lymphocyte, Checkpoint blockade, Exome, Vaccine, Next-generation sequencing, Omics

## Abstract

T cells can mediate remarkable tumor regressions including complete cure in patients with metastatic cancer. Genetic alterations in an individual’s cancer cells (the mutanome) encode unique peptides (m-peptides) that can be targets for T cells. The recent advances in next-generation sequencing and computation prediction allows, for the first time, the rapid and affordable identification of m-peptides in individual patients. Despite excitement about the extended spectrum of potential targets in personalized immunotherapy, there is no experience or consensus on the path to their successful clinical application. Major questions remain, such as whether clinical responses to cytokine therapy, T cell transfer, and checkpoint blockade are primarily mediated by m-peptide-specific reactivity, whether m-peptides can be effectively used as vaccines, and which m-peptides are most potently recognized. These and other technological, immunological and translational questions will be explored during a 1-day Workshop on Personalized Cancer Immunotherapy by the Society for Immunotherapy of Cancer, directly before the Annual Meeting, on November 7, 2013 at the National Harbor, MD near Washington, DC.

## Introduction

### The cancer mutanome: is it important?

**Figure 1:**
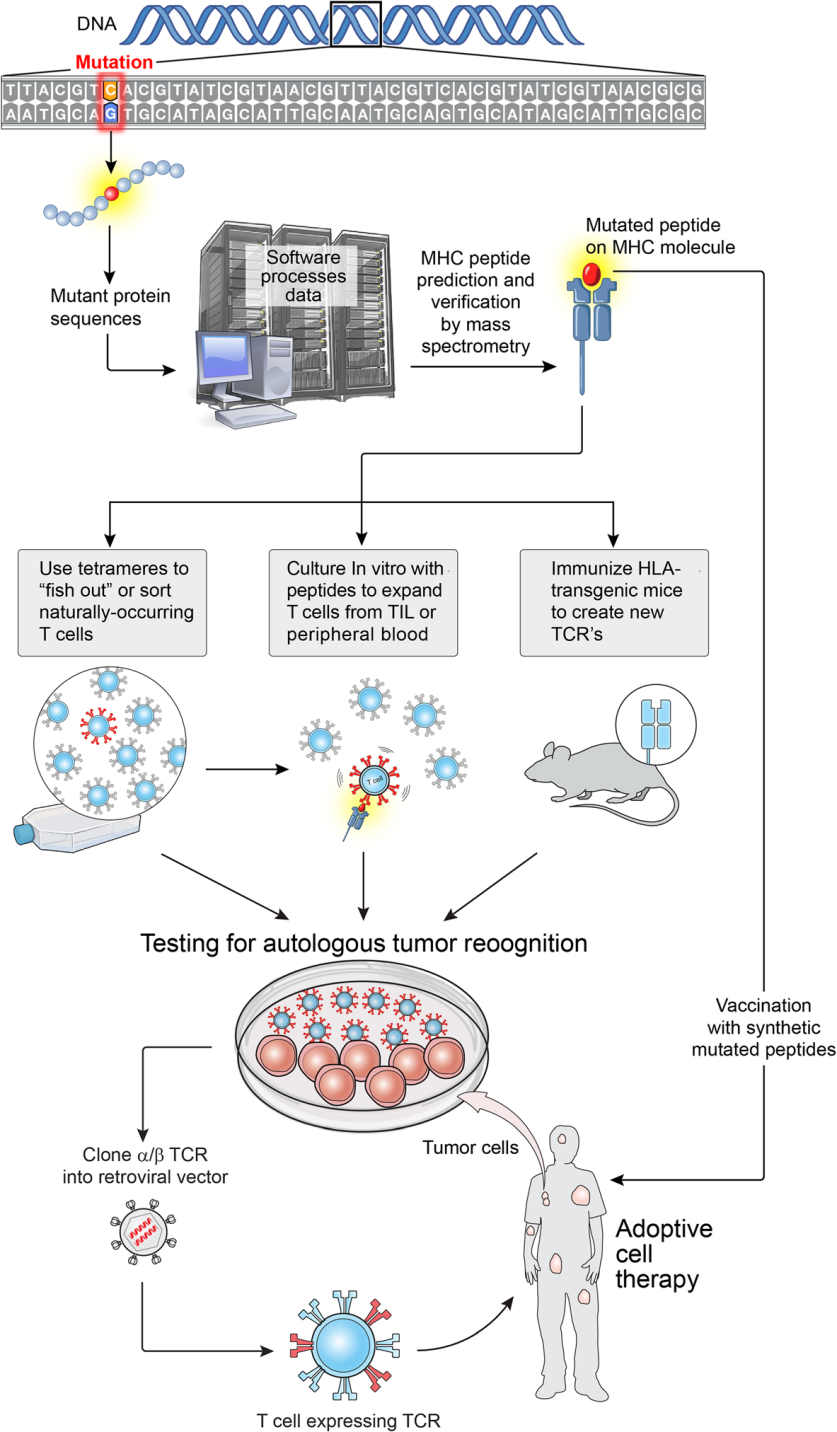
**Highly personalized medicine.** Inexpensive and highly available DNA sequencing can revolutionize cancer immunotherapy by enabling highly personalized approaches involving the identification of new tumor-associated antigens. The expressed genes from a patient’s tumor can be sequenced to identify candidate mutant T cell epitopes. Relevant epitopes that could potentially bind to any given patient’s HLA molecules could be predicted using peptide prediction algorithms (*e*.*g*. http://www.syfpeithi.de/bin/MHCServer.dll/EpitopePrediction.htm. Or http://www-bimas.cit.nih.gov/molbio/hla_bind). If peptides derived from mutant proteins are found to capable of forming new HLA-restricted target structures, the candidate peptides can be used in one of at least several ways: 1) “fish out” or sort cells for relevant antigens (such as those specific for driver oncogenes) using tetramer like reagents; 2) use the candidate peptides to stimulate T cell clonotypes already present in a patient’s tumor or in their peripheral blood; 3) use antigens to elicit new T cell receptors in mice that are transgenic for human MHC molecules; and 4) to immunize patients against antigens. If the T cells generated are specific for a patient’s tumor, they can be expanded and adoptively transferred if they are of human origin, or used as a source of TCR for gene engineering approaches.

Peptides encoded by mutated genes in cancer cells have long been recognized as potential T cell targets, yet they were not pursued for personalized cancer therapy due to time and cost constraints on their identification. The recent arrival of next-generation sequencing and bioinformatics approaches allows, for the first time, the rapid and affordable elucidation of an individual cancer patient’s genome, exome, epigenome and transcriptome at the single nucleotide level [1]. This in turn enables the identification of patient-specific omic alterations that can function as unique therapeutic targets such as neoantigens [2-8] (Figure 1). The collective of an individual patient’s tumor-specific alterations and mutations, the so-called mutanome, thus encodes patient-specific antigens that are different from “shared” antigens, which are expressed in tumors from multiple patients and are typically normal, non-mutated self-proteins. In particular, mutanome-encoded peptides (hereafter called m-peptides) may evoke a more vigorous T cell response due to a lack of thymic tolerance against them, and this immunity may be restricted to tumors, since the mutated gene product is only expressed in tumors [9].

These assumptions have not been rigorously tested, and our understanding of, for example, m-peptide-specific peripheral tolerance and T cell cross-reactivity with wild-type peptides is limited at best. Yet several very exciting recent reports on the power of immunity against m-peptides to shrink tumors in mice and patients suggest the importance of understanding the biology of m-peptides and their application in immunotherapy. For example, a tantalizing possibility is that the remarkable clinical response rates to adoptively transferred T cells, and to CTLA-4 and/or PD(L)-1 checkpoint blockade, are mediated in part, or even primarily, by m-peptide-specific T cells [10,11]. M-peptide-specific T cells present before treatment may be a required substrate for successful therapy with cytokines and checkpoint blockade. Thus, an exciting possibility is that peptide-based vaccination [12,13] with m-peptides could induce tumor-specific T cells that are lacking in patients who have failed to respond to checkpoint blockade, and convert these patients into responders. M-peptides may also prove to be major natural targets for tumor-specific TIL [6], and could be used for *ex vivo* expansion of patient-derived T cells (TIL or PBMC) before adoptive T cell therapy. In addition, m-peptides could be efficiently targeted with TCR-transduced T cells. Finally, m-peptides may be useful in immune monitoring, to evaluate whether m-peptide-specific immunity correlates with response or disease recurrence after immunotherapy. Thus, understanding mutanome-encoded m-peptides as a target for anti-tumor T cells is a new frontier for cancer immunotherapy.

## Review

### Recent studies on mutanome-specific anti-tumor immunity

The systematic study of the mutanome has only just begun. Importantly, it appears that different cancer types and histologies harbor different numbers of mutations. Those with high numbers of mutation include cutaneous melanoma, smoking-induced lung cancer, and colon cancer, in particular hypermutators [9,14]. This is likely related to the high exposure of the respective cells of origin to mutagenic and inflammatory stimuli: UV light, cigarette smoke and food-contained mutagenic and inflammatory compounds. It is interesting that melanoma and lung cancer both respond particularly well to PD-1 immunotherapy, possibly implying that m-peptides are particularly involved in the therapeutic T cell response to this therapy [15,16].

In mice, methylcholanthrene-induced sarcomas likewise harbor a large number of mutations, and a subset of these tumors are particularly immunogenic: they will grow in immunodeficient but not in immunocompetent mice. Matsushita *et al*. analyzed the mutanome of one such immunogenic tumor by exome sequencing and MHC binding algorithms and identified a point mutation in the spectrin-β2 gene that resulted in an m-peptide with greatly increased MHC Class I binding. This peptide proved to be a dominant tumor antigen that caused the complete, spontaneous, CD8^+^ T cell-mediated regression of this tumor in immunocompetent mice [14]. Using a similar approach, Castle *et al*. analyzed the mutanome of the widely used B16 melanoma cell line and tested 50 MHC-binding m-peptides, 16 of which were immunogenic and 11 of which preferentially recognized the mutant peptide over the wild-type counterpart. Importantly, they showed that vaccination with 2 of those suppressed the growth suppression of established B16 melanomas [17]. Robbins *et al*. similarly studied melanomas from 3 patients who responded to therapy with *ex*-*vivo* expanded autologous tumor-infiltrating lymphocytes (TIL), and identified a total of 7 unique m-peptides that appeared to be processed and presented by autologous tumor cells and were recognized by the autologous TIL [11].

These three studies used exome sequencing and computer algorithm-guided peptide epitope prediction to identify MHC Class I-binding m-peptides that were processed and presented by tumor cells and recognized by tumor-specific T cells, thus validating the approach. However, other approaches exist, such as the direct elution of peptides from the surface of tumor cells, or even from circulating tumor-derived HLA complexes, and their subsequent identification by microcapillary chromatography/tandem mass spectrometry [18]. In combination with tumor exome data, or after *in silico* subtraction of peptides isolated from normal cells, tumor-expressed m-peptides can be identified, including peptides that undergo post-translational processing, and that would not be identified using genetic approaches alone.

### Mutanome-based personalized immunotherapy: open questions

With the technology to efficiently mine the mutanome in a high-throughput fashion only just becoming available, many questions remain regarding its application in personalized immunotherapy. Some of these are:

1. What are the possible and most important uses of m-peptides?

2. Are m-peptides superior to shared/self-peptides as therapeutic targets for anti-tumor T cells?

3. What fraction of m-peptides is actually processed and presented in the context of MHC molecules on tumor cells?

4. What fraction of these peptides evokes a T cell response in the host (spontaneously or after vaccination)?

5. How frequently do T cells against m-peptides cross-react with the corresponding wild-type peptides expressed on normal cells?

6. Are m-peptides the dominant peptides recognized in spontaneous tumor-specific immune responses?

7. Can m-peptide-based vaccines induce therapeutic immune responses, alone or as part of combination therapies?

8. Are m-peptides the dominant peptides recognized in therapy-induced immune responses (*e*.*g*. checkpoint blockade, TIL, IL-2, IFN-α, radiation, immunogenic chemotherapies).

9. What “pipeline” allows the shortest time from fresh tumor sample to validated m-peptide?

10. What are the (dis)advantages of sequencing exomes *vs*. RNA?

11. What are the best approaches for filtering the numerous sequencing errors before declaring a somatic mutation?

12. What peptide prediction algorithms are best at predicting MHC-binding peptides from exome data?

13. What is the impact of the immunoproteasome, peptide splicing, and post-translational modification on the actual expression of predicted m-peptides on tumor cells?

14. What are safety and regulatory issues in designing personalized cancer vaccines against never-before targeted, patient-specific antigens?

## Conclusions

### The 2013 society for immunotherapy of cancer workshop on personalized immunotherapy

The mining of the mutanome has just begun, and many questions regarding methodology, application and future opportunities remain open and ready for debate. With many groups currently engaging this area, it is a prime time to come together and share data, viewpoints and best practices, forge collaborations, and chart a course for the immediate future. The Society for Immunotherapy of Cancer invites you to attend their Workshop on Personalized Immunotherapy, directly before the Annual Meeting, on Nov 7, 2013 at the National Harbor, MD near Washington, DC. Specifics can be found at the Society website at http://www.sitcancer.org/2013/workshop. Abstracts and posters are welcome through August 26, 2013, and platform presentations will be selected.

## Abbreviations

TIL: Tumor-infiltrating lymphocytes; PBMC: Peripheral blood mononuclear cells; TCR: T cell receptor; MHC: Major histocompatibility complex; HLA: Human leukocyte antigen.

## Competing interests

The authors declare that they have no competing interests.

## Authors’ contributions

WWO, EW, FMM, HGR and NPR conceived the Workshop idea and topics and participated in writing the manuscript. All authors read and approved the final manuscript.
